# ESBL-Producing *Moellerella wisconsensis*—The Contribution of Wild Birds in the Dissemination of a Zoonotic Pathogen

**DOI:** 10.3390/ani12030340

**Published:** 2022-01-30

**Authors:** Zoi Athanasakopoulou, Marina Sofia, Alexios Giannakopoulos, Konstantinos Papageorgiou, Dimitris C. Chatzopoulos, Vassiliki Spyrou, Evanthia Petridou, Efthymia Petinaki, Charalambos Billinis

**Affiliations:** 1 Faculty of Veterinary Science, University of Thessaly, 43100 Karditsa, Greece; zathanas@uth.gr (Z.A.); msofia@uth.gr (M.S.); algiannak@uth.gr (A.G.); 2 Faculty of Public and One Health, University of Thessaly, 43100 Karditsa, Greece; pgkostas@yahoo.gr (K.P.); dchatzopoulos@uth.gr (D.C.C.); 3 Faculty of Veterinary Medicine, Aristotle University of Thessaloniki, 54124 Thessaloniki, Greece; epetri@vet.auth.gr; 4 Faculty of Animal Science, University of Thessaly, 41110 Larissa, Greece; vasilikispyrou@uth.gr; 5 Faculty of Medicine, University of Thessaly, 41500 Larissa, Greece; petinaki@uth.gr

**Keywords:** *Moellerella wisconsensis*, ESBL, multidrug resistance, Enterobacteriaceae, wild birds, Greece, CTX-M-1

## Abstract

**Simple Summary:**

*Moellerella wisconsensis* is a potentially zoonotic pathogen that has sporadically been isolated from animals and humans. In the present study, we describe the occurrence of the organism among 445 wild bird and 2000 human fecal samples, in the context of an investigation regarding antimicrobial resistant bacteria in Greece. According to our results, 0.9% (*n* = 4) of the examined wild birds were found to be colonized by *M. wisconsensis*, while no human was a carrier of the bacterium. Out of the total number of four *M. wisconsensis* strains that we detected, three presented resistance to 3rd generation cephalosporins, were phenotypically confirmed to produce extended spectrum beta lactamases (ESBLs) and harbored *bla*_CTX-M-1_. Resistance to tetracyclines, aminoglycosides and trimethoprim/sulfamethoxazole was additionally detected in three, two and one of the ESBL isolates, respectively. This is the first report that presents the dissemination of *M. wisconsensis* in wild bird from Greece and describes CTX-M-1 production in multidrug resistant wild birds’ isolates of this bacterial species.

**Abstract:**

*Moellerella wisconsensis* is an Enterobacteriaceae with unclarified dispersion and pathogenicity. During an ongoing investigation about antimicrobial resistance in Greece, the occurrence of *M. wisconsensis* was evaluated among wild birds and humans. A total of 445 wild bird and 2000 human fecal samples were collected and screened for the presence of the organism. Subsequently, all *M. wisconsensis* strains were phenotypically and molecularly characterized regarding their antimicrobial resistance characteristics. Four *M. wisconsensis* were isolated from a common pheasant (*Phasianus colchicus*), two Eurasian magpies (*Pica pica*) and a great white-fronted goose (*Anser albifrons*). Among these four strains, the three latter presented resistance to 3rd generation cephalosporins, were phenotypically confirmed to produce ESBLs and were found to harbor *bla*_CTX-M-1_. The three ESBL isolates additionally exhibited resistance to tetracyclines, while resistance to aminoglycosides was detected in two of them and to trimethoprim/sulfamethoxazole in one. No *Moellerella wisconsensis* strains were retrieved from the human samples tested. This is the first report that provides evidence of *M. wisconsensis* dissemination among wild birds in Greece, describing CTX-M-1 production in multidrug resistant wild birds’ isolates of this bacterial species.

## 1. Introduction

The genus *Moellerella* includes a monophyletic species, *Moellerella wisconsensis* (*M. wisconsensis*), which was named after Wisconsin USA, where the majority of the earliest detected strains originated from [1]. *M. wisconsensis*, previously designated as Enteric Group 46, is a gram negative, nonmotile, facultative anaerobic and fermentative bacillus. It is a member of the Enterobacteriaceae family, presents the general characteristics of the family, and is taxonomically mostly related to *Providencia* spp. *M. wisconsensis* additionally exhibits intrinsic resistance to colistin and polymyxin B, which is considered to be a key feature for its identification [1,2].

Despite that almost forty years have passed since the first detection of *M. wisconsensis* in 1984, its exact distribution and potential pathogenicity remain widely unknown. The bacterium has been isolated from human clinical specimens and has been implicated in cases of gastroenteritis, diarrhea, cholecystitis, bacteremia, peritonitis and urinary tract infections [1,2,3,4,5,6,7]. Additionally, it has been retrieved from both domestic and wild animals, as well as from insects and parasites, and has been identified as the causative agent of animal disease [8,9,10,11,12]. However, its role in the etiology of clinical conditions has not been fully elucidated, given the infrequent human and animal colonization. Notably, the scarcity of reported infections caused by *M. wisconsensis* has been partly ascribed to its misidentification as *Escherichia coli* (*E. coli*) or *Klebsiella pneumoniae* subsp. *ozaenae* [13]. Subsequently, *M. wisconsensis* can be regarded as a rare, though potentially underestimated, opportunistic, zoonotic pathogen with virulence that remains to be clarified.

According to the Centers for Disease Control and Prevention, Enterobacteriaceae that produce extended spectrum beta lactamases (ESBL) are classified as a serious threat for healthcare settings and the community [14]. ESBL carriage in wild birds is perceived to be a result of a spill-over phenomenon through environmental pollution with human or domestic animal strains. Wild birds can, thus, either become colonized by already resistant bacteria via their contact with human waste, sewage, and livestock manure or acquire ESBL genes horizontally from resistant isolates that occur in their environment [15]. ESBL-producing Enterobacteriaceae (ESBL-PE) seem to be particularly disseminated among aquatic associated, omnivorous and synanthropic species, while migratory wild birds are of the most concern for their further dispersal [16,17,18].

The aim of the present study was to describe the occurrence of *M. wisconsensis*, a rather rare Enterobacteriaceae, among fecal samples of wild birds and humans in Greece and to present its antimicrobial resistance profile.

## 2. Materials and Methods

### 2.1. Sample Collection

During an ongoing investigation regarding antimicrobial resistant bacteria in Greece, non-duplicated fecal samples were collected from 445 wild birds as well as from 2000 patients of the University Hospital of Larissa, between January 2019 and June 2021.

Wild birds were captured using Larsen and Australian type traps, as well as modified bird catching nets, sampled directly from the cloaca and immediately released, according to the prerequisites of the Greek Legislation. Alternatively, samples were collected after identifying the wild bird species, scaring off the bird, and then sampling the freshly dropped feces. Specimens were obtained using sterile cotton swabs and were placed in Amies transport medium (Transwab^®^ Amies, Leicester, UK). Transportation was performed under refrigeration and the samples arrived in the laboratory within 48 h of their initial collection. The sampling sites of wild birds were located in a variety of habitats, including urban and suburban areas, wetlands, pastures, scrubs/meadows, forests, agroforestry formations and agricultural areas. The exact sampling position of each wild bird was recorded using handheld Global Positioning System (GPS) units (GPSMAP 62s, Garmin Ltd., Southampton, UK).

Human samples originated from the University Hospital of Larissa, a tertiary care 600-bed hospital in Thessaly region (Central Greece), which serves a population of approximately 1,000,000 inhabitants. All patients’ fecal swabs were retrieved for routine cultures at the time of admission to the hospital and prior to the administration of any antibiotic therapy.

### 2.2. Isolation, Identification and Antimicrobial Resistance Phenotype of Moellerella wisconsensis

Swabs were directly streaked onto both MacConkey agar and ESBL selective agar (CHROMID^®^, BioMérieux, Marcy l’Etoile, France). One colony per plate was selected and further processed. Identification and antimicrobial susceptibility testing of the obtained strains were performed using the Vitek-2 system (BioMérieux, Marcy l’Etoile, France) and the GN ID and AST-GN96 cards, respectively, as previously described [19]. Isolates were characterized as multidrug resistant (MDR) when they presented diminished susceptibility to at least one agent of more than three classes of antibiotics.

Bacterial DNA of all the isolates that were identified as *M. wisconsensis* was extracted from overnight cultures using the PureLink^TM^ Genomic DNA Mini Kit (Invitrogen, Darmstadt, Germany), according to the manufacturer’s instructions for Gram-negative bacteria. Subsequently, the identity of the isolates was verified by amplification of a 760 bp fragment of the 16S rDNA via PCR [20] (Table 1 and sequencing of the amplicons (3730xl DNA Analyzer, Applied Biosystems, Foster City, CA, USA).

### 2.3. Phylogenetic Analysis

The 16S rDNA sequences of the strains obtained in the present study and of all the *M. wisconsensis* sequences available in GenBank (*n* = 20) were aligned by ClustalW. The phylogenetic tree was constructed with the Neighbor-Joining method [21] and the evolutionary distances were computed using the Kimura 2-parameter [22]. A bootstrap resampling analysis for 1000 replicates was performed to estimate the confidence of tree topologies [23]. Analyses were conducted in MEGA 11 [24].

### 2.4. Phenotypic Evaluation and Molecular Confirmation of ESBL Production

According to the results of susceptibility testing, *M. wisconsensis* isolates that presented resistance to 3rd generation cephalosporins (cefoperazone, ceftiofur) were phenotypically screened for ESBL production by the double-disk synergy test (DDST) [25]. Isolates that presented a positive DDST were further subjected to molecular confirmation. Simplex PCRs were performed for the amplification of genes encoding the most common types of ESBLs, namely CTX-M, TEM and SHV, using the primers described by *Dandachi I*. et al. [26] (Table 1). In all the assays, sterile distilled water served as negative control, while confirmed ESBL-producing Enterobacteriaceae from our strains’ collection were used as positive control. Post-amplification products were visualized on 2% agarose gel electrophoresis. The PCR products were purified and analyzed by sequencing (3730xl DNA Analyzer, Applied Biosystems).

## 3. Results

A total number of four (4/445; 0.9%) wild birds were found to be colonized with *M. wisconsensis*. The strains were identified according to their biochemical characteristics (Appendix A, Table A1). In particular, *M. wisconsensis* was isolated from the fecal samples of a common pheasant (*Phasianus colchicus*) originating from Atalanti island, two Eurasian magpies (*Pica pica*) from Lake Karla and a great white-fronted goose (*Anser albifrons*) from Lake Pamvotis. *M. wisconsensis* was not detected in any of the tested human samples.

**Figure 1:**
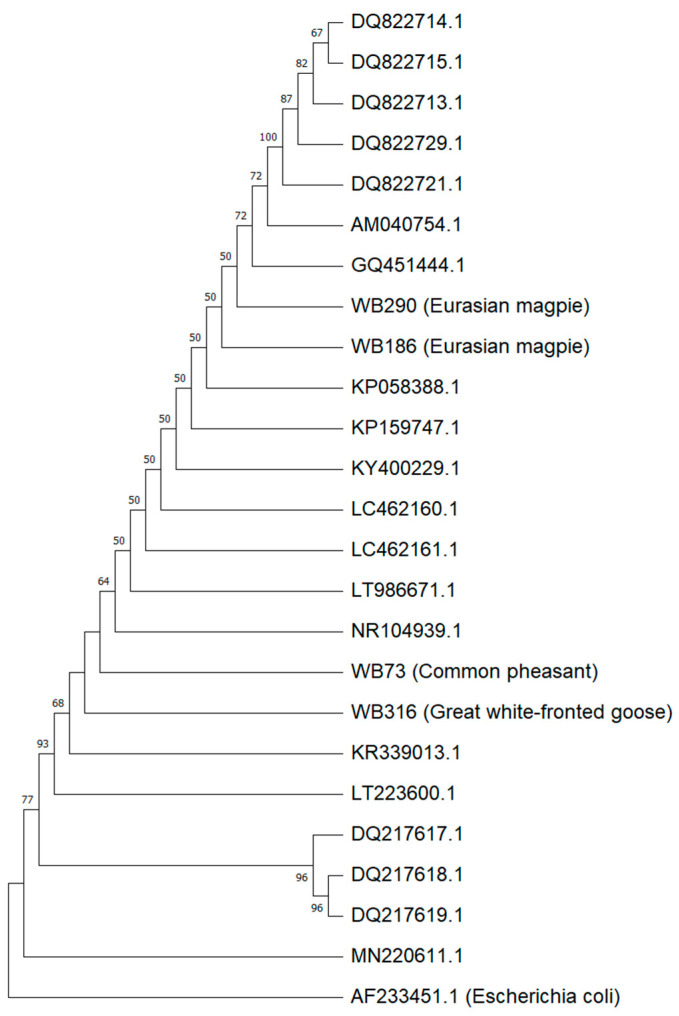
Phylogenetic tree constructed with Neighbor-Joining method by using the four 16S rDNA sequences of Greek *M. wisconsensis* isolates (WB73, WB186, WB290, WB316; in red boxes) and 20 *M. wisconsensis* sequences retrieved from the GenBank database. *E. coli* strain AF233451.1 was used as the outgroup. Bootstrap values (expressed as percentages of 1000 replications) are shown at the branch points; only values over 50% are indicated.

Sequence analysis of the 16S rDNA confirmed the presence of *M. wisconsensis* in all four wild birds’ samples. The four Greek isolates were aligned on a region of 675 nucleotides with 20 isolates from humans, animals and insects that had previously been deposited in GenBank and were found to present over 99.9% similarity with them. The evolutionary relationships between the 24 sequences were described by a Neighbor-Joining tree (Figure 1).

All the isolates presented resistance to polymixin B. The strain isolated from the common pheasant was obtained from MacConkey agar and was additionally resistant to cefalexin. The remaining three strains were obtained from ESBL selective agar and presented the ESBL phenotype, being resistant to penicillins (ampicillin) and 1st to 4th generation cephalosporins. These three ESBL strains were also resistant to tetracyclines, while two of them, one from a magpie and the one from goose, exhibited reduced susceptibility to aminoglycosides. The aminoglycosides resistant magpie strain was further resistant to trimethoprim/sulfamethoxazole.

Molecular screening for ESBL encoding genes in the three phenotypic ESBL producers revealed that they all carried *bla*_CTX-M-1_, while *bla*_TEM_ and *bla*_SHV_ were not detected in any isolate.

The characteristics of the four *M. wisconsensis* isolates are summarized in Table 2.

## 4. Discussion

In the present study, we detected carriage of *M. wisconsensis* by 0.9% of the sampled wild birds and specifically by a common pheasant, two magpies and a great white-fronted goose and we describe the presence of three MDR ESBL-producing *M. wisconsensis* for the first time in Greece. Notably, the organism was not identified in the examined human samples, confirming that *M. wisconsensis* is a rare clinical isolate [2].

Even though *M. wisconsensis* is probably part of the normal gastrointestinal microbiota, its natural habitat is speculated to be associated with the environment and particularly with water [1]. Our results support this claim, since *M. wisconsensis* was detected in four wild birds’ samples that lived in proximity to three different aquatic environments (Atalanti island, lake Karla, lake Pamvotis) but not in any samples collected from wild birds inhabiting other types of environments.

The isolation of *M. wisconsensis* from wild animals has sparsely been reported in previous studies. In the USA, *Bangert R*. and colleagues have identified fecal carriage of the bacterium by 9% of the examined captive raptors (Falconiformes and Strigiformes), which were on a diet primarily consisting of commercially prepared chicken [27]. Given the fact that these wild birds were under captivity, it cannot be inferred whether the presence of the bacterium represented a natural colonization or was a result of their interaction with humans. In the same country, *M. wisconsensis* has also been retrieved from the oral secretions of a wild raccoon, which was suggested to be a zoonotic reservoir of the organism [10], while, in Italy, it has been isolated from fecal samples of foxes, mustelids and a wolf [28].

Three of the isolated *M. wisconsensis*, from the two magpies and the goose, presented diminished susceptibility to at least one agent of more than three antimicrobial categories and were subsequently characterized as multidrug resistant [29]. The fact that these strains harbored an ESBL gene could indicate a human or livestock to wildlife transmission of either the strain itself or of its resistance determinants. However, ESBL-producing *M. wisconsensis* was neither detected in the human samples that we examined nor has, to date, been reported from Greece in human or other animal samples. Thus, the second hypothesis seems more plausible. Nevertheless, further studies are required to fully elucidate either of the speculations. *bla*_CTX-M-1_ was the only ESBL gene that we detected, a finding that is in agreement with data on the current molecular epidemiology of ESBL-producing Enterobacteriaceae among both domestic and wild animals worldwide [30,31,32]. A sole former study has described ESBL carriage in a single *M. wisconsensis* isolate that was retrieved from flies trapped in the surrounding area of a hospital in Ethiopia [33]. In that study, though, *M. wisconsensis* carried the *bla*_TEM_ ESBL gene (personal communication with Dr. Tufa, T.B.).

*Stock I.* et al. have previously detailed the natural antibiotic susceptibility of *M. wisconsensis*. According to their findings, the species presents natural sensitivity to aminoglycosides, fluoroquinolones, folate-pathway inhibitors (trimethoprim/sulfamethoxazole) and tetracyclines [13]. The resistance profile of the three MDR isolates in our study is therefore, presumably, a result of acquired antimicrobial resistance mechanisms. Genes encoding ESBLs are most commonly located on transferable plasmids, rather than the bacterial genome, which frequently also carry resistance determinants for various other antimicrobial classes, including the aforementioned ones [34]. This fact, along with the ability of Enterobacteriaceae to acquire multiple plasmids, as well as to mutate against antimicrobials could explain the reported multidrug resistance in our strains [35].

Carriage of MDR, ESBL-producing Enterobacteriaceae from magpies is probably associated with the birds synanthropic, omnivorous and scavenging behavior, as has previously been described for *E. coli* strains isolated from the species [19,36]. Adult magpies are sedentary, while the dispersion of juveniles is limited and does not extend beyond 30–40 km from the place of birth. Lake Karla, however, where the two ESBL *M. wisconsensis* from magpies were detected, is a site of great importance for migratory and overwintering waterbirds and foraging raptors in Greece. Subsequently, these birds could obtain the strains and further contribute to their environmental dissemination across long distances during migration. Correspondingly, the great white-fronted goose, that was also found to be colonized by a MDR ESBL-producing *M. wisconsensis*, is a migratory species that conducts long and short distance migration with a potential to diffuse both *M. wisconsensis* and *bla*_CTX-M__-1_. This bird most probably acquired the resistant strain or the respective resistance determinants from its habitat, since lake Pamvotis is known to be impaired by pollutants from sewage [37].

## 5. Conclusions

In conclusion, this study revealed wild birds’ colonization with *M. wisconsensis* in Greece. Three out of the four isolates presented a multidrug resistant, ESBL-producing phenotype and harbored *bla*_CTX-M-1_. Our findings underline the potential role of wild birds in both the spread of *M. wisconsensis* and the dissemination of *bla*_CTX-M-1_.

## Figures and Tables

**Table 1:** Primer sequences, amplicon size and optimal annealing temperature of each simplex PCR performed in the study.

Target	Primer Sequence (5′-3′)	Amplicon Size (bp)	Annealing Temperature (°C)
*Moellerella wisconsensis* 16S rDNA	F: CTC GTT GCG GGA CTT AAC	760	60
	R: ACT CCT ACG GGA GGC AGC A		
*bla*_CTX-M_	F: ATG TGC AGY ACC AGT AAR GTK ATG GC	593	60
	R: TGG GTR AAR TAR GTS ACC AGA AYC AGC GG		
*bla*_SHV_	F: CTT TAT CGG CCC TCA CTC AA	327	60
	R: AGG TGC TCA TCA TGG GAA AG		
*bla*_TEM_	F: CGC CGC ATA CAC TAT TCT CAG AAT GA	445	62
	R: ACG CTC ACC GGC TCC AGA TTT AT		

**Table 2:** Origin, antimicrobial resistance profile and ESBL genes of the *M. wisconsensis* isolates.

Strain ID	Wild Bird Species	Region	Regional Unit	Antimicrobial Resistance Phenotype	ESBL Genotype
WB73	Common pheasant (*Phasianus colchicus*)	Atalanti island	Fthiotida	CEX, PMB	-
WB186	Eurasian magpie (*Pica pica*)	Lake Karla	Magnesia	AMP, CEX, CF, CEP, CEF, CEQ, TET, PMB	*bla*_CTX-M-1_
WB290	Eurasian magpie (*Pica pica*)	Lake Karla	Magnesia	AMP, CEX, CF, CEP, CEF, CEQ, GEN, TET, PMB, SXT	*bla*_CTX-M-1_
WB316	Great white-fronted goose (*Anser albifrons*)	Lake Pamvotis	Ioannina	AMP, CEX, CF, CEP, CEF, CEQ, GEN, NEO *, TET, PMB	*bla*_CTX-M-1_

AMP—ampicillin, CEX—cefalexin, CF—cefalotin, CEP—cefoperazone, CEF—ceftiofur, CEQ—cefquinome, GEN—gentamicin, NEO—neomycin, TET—tetracycline, PMB—polymixin B, SXT—trimethoprim/sulfamethoxazole, * intermediate resistance, “-”—the isolate did not harbor an ESBL gene.

## Data Availability

Most data for this study are presented within the manuscript. The remaining data are available on request from the corresponding author. The data are not publicly available as they are part of the PhD thesis of the first author, which has not yet been examined, approved and uploaded in the official depository of PhD theses from Greek Universities.

## Appendix A

**Table A1:** Biochemical characteristics of the four *Moellerella wisconsensis* strains detected in the study.

Biochemical Reaction	WB73	WB186	WB290	WB316
Probability of correct identification	99%	99%	99%	99%
Ala-Phe-Pro-Arylamidase	-	-	-	-
Adonitol	+	+	+	+
L−Pyrrolydonyl−Arylamidase	-	-	-	-
L−Arabitol	-	-	-	-
D−Cellobiose	-	-	-	-
Beta−Galactosidase	+	+	+	+
H2S Production	-	-	-	-
Beta−N−Acetyl−Glucosaminidase	-	-	-	-
Glutamyl Arylamidase pNA	-	-	-	-
D−Glucose	+	+	+	+
Gamma−Glutamyl−Transferase	-	-	-	-
Fermentation/Glucose	+	+	+	+
Beta−Glucosidase	-	+	(-)	+
D−Maltose	-	-	-	-
D−Mannitol	-	-	-	-
D−Mannose	+	+	+	+
Beta−Xylosidase	-	-	-	-
BETA−Alanine arylamidase pNA	-	-	-	-
L−Proline Arylamidase	-	-	-	-
Lipase	-	-	-	-
Palatinose	-	-	-	-
Tyrosine Arylamidase	+	+	+	+
Urease	-	-	-	-
D−Sorbitol	-	-	-	-
Saccharose/Sucrose	+	+	+	+
D−Tagatose	-	-	-	-
D−Trehalose	-	-	-	-
Citrate (Sodium)	+	+	+	+
Malonate	-	-	-	-
5−Keto−D−Gluconate	-	-	-	-
L−Lactate Alkalinisation	-	-	-	-
Alpha−Glucosidase	-	-	-	-
Succinate alkalinisation	+	+	+	+
Beta−N−Acetyl−Galactosaminidase	-	-	-	-
Alpha−Galactosidase	(+)	+	+	+
Phosphatase	+	(-)	(+)	+
Glycine Arylamidase	-	-	-	-
Ornithine Decarboxylase	-	-	-	-
Lysine Decarboxylase	-	-	-	-
L−Histidine assimilation	-	-	-	-
Coumarate	+	+	+	+
Beta−Glucoronidase	-	-	-	-
O/129 Resistance (comp.vibrio.)	-	+	+	-
Glu−Gly−Arg−Arylamidase	-	-	-	-
L−Malate assimilation	-	-	-	-
Ellman	-	-	-	-
L−Lactate assimilation	-	-	-	-

“+”— positive reaction, “-”— negative reaction, reactions that appear in parentheses are indicative of weak reactions that are too close to the test threshold.
